# The impact of 2D cine MR imaging parameters on automated tumor and organ localization for MR-guided real-time adaptive radiotherapy

**DOI:** 10.1088/1361-6560/aae74d

**Published:** 2018-11-22

**Authors:** Martin J Menten, Martin F Fast, Andreas Wetscherek, Christopher M Rank, Marc Kachelrieß, David J Collins, Simeon Nill, Uwe Oelfke

**Affiliations:** 1 Joint Department of Physics at The Institute of Cancer Research and The Royal Marsden NHS Foundation Trust, London, United Kingdom; 2 Department of Radiation Oncology, The Netherlands Cancer Institute, Amsterdam, Netherlands; 3 Medical Physics in Radiology, German Cancer Research Center (DKFZ), Heidelberg, Germany; martin.menten@icr.ac.uk

**Keywords:** MR-linac, MR-guided radiotherapy, real-time adaptive radiotherapy, 2D cine MR imaging

## Abstract

2D cine MR imaging may be utilized to monitor rapidly moving tumors and organs-at-risk for real-time adaptive radiotherapy. This study systematically investigates the impact of geometric imaging parameters on the ability of 2D cine MR imaging to guide template-matching-driven autocontouring of lung tumors and abdominal organs.

Abdominal 4D MR images were acquired of six healthy volunteers and thoracic 4D MR images were obtained of eight lung cancer patients. At each breathing phase of the images, the left kidney and gallbladder or lung tumor, respectively, were outlined as volumes of interest. These images and contours were used to create artificial 2D cine MR images, while simultaneously serving as 3D ground truth. We explored the impact of five different imaging parameters (pixel size, slice thickness, imaging plane orientation, number and relative alignment of images as well as strategies to create training images). For each possible combination of imaging parameters, we generated artificial 2D cine MR images as training and test images. A template-matching algorithm used the training images to determine the tumor or organ position in the test images. Subsequently, a 3D base contour was shifted to the determined position and compared to the ground truth via centroid distance and Dice similarity coefficient.

The median centroid distance between adapted and ground truth contours was 1.56 mm for the kidney, 3.81 mm for the gallbladder and 1.03 mm for the lung tumor (median Dice similarity coefficient: 0.95, 0.72 and 0.93). We observed that a decrease in image resolution led to a modest decrease in localization accuracy, especially for the small gallbladder. However, for all volumes of interest localization accuracy varied substantially more between subjects than due to the different imaging parameters.

Automated tumor and organ localization using 2D cine MR imaging and template-matching-based autocontouring is robust against variation of geometric imaging parameters. Future work and optimization efforts of 2D cine MR imaging for real-time adaptive radiotherapy is needed to characterize the influence of sequence- and anatomical site-specific imaging contrast.

## 1. Introduction

Respiratory, cardiac and gastrointestinal induced motion causes substantial changes in patient anatomy over the course of a single radiotherapy fraction (Langen and Jones 2001, Seppenwoolde *et al* 2002, Bussels *et al* 2003, Kitamura *et al* 2003). This may lead to underdosage of the tumor and additional radiation exposure of critical organs (Keall *et al* 2006). Real-time adaptive radiotherapy aims at continuously monitoring the position of the tumor and nearby organs and adjusting the radiation delivery accordingly, for example by gating the treatment beam (Shirato *et al* 2000) or changing the beam’s position and shape using a multileaf collimator (Keall *et al* 2001, Tacke *et al* 2010, Fast *et al* 2014, Keall *et al* 2014b). The prospect of guiding such real-time adaptive techniques with magnetic resonance (MR) imaging has been one of the driving forces behind the development of hybrid MR imaging/radiotherapy machines (Fallone *et al* 2009, Raaymakers *et al* 2009, Keall *et al* 2014a, Mutic and Dempsey 2014). The on-board MR imaging is able to provide images with high soft-tissue contrast during radiation delivery without the need for implanted fiducial markers or an additional imaging dose.

Currently, it is not possible to acquire, reconstruct and postprocess 3D MR images of sufficient spatial coverage and resolution at an imaging rate adequate to track anatomical structures whose movement is primarily governed by respiratory motion. Instead, 2D cine MR imaging, which is able to survey one or multiple 2D imaging planes in real-time, may be harnessed to monitor rapidly moving tumors and organs-at-risk. Several groups have developed autocontouring algorithms to outline the tumor visible in 2D MR images (Cervino *et al* 2011, Shi *et al* 2014, Paganelli *et al* 2015, Yun *et al* 2015, Bourque *et al* 2016, Feng *et al* 2016, Mazur *et al* 2016, Fast *et al* 2017, Yip *et al* 2018), with the accuracy of these algorithms approaching inter-observer variability (Shi *et al* 2014, Fast *et al* 2017, Yip *et al* 2018). However, localization accuracy in a 2D plane does not necessarily translate into usefulness for determining an anatomical structure’s position and extent in three dimensions. The volume of interest may deform, shift perpendicularly to the plane or move out of it entirely. For this reason, others have investigated how to best position, orientate and utilize 2D cine MR images for tumor and organ localization (Bjerre *et al* 2013, Tryggestad *et al* 2013, Brix *et al* 2014, Ipsen *et al* 2016, Bourque *et al* 2018). These studies were mostly based on heuristic approaches, tested only a small number of imaging strategies and often lacked a 3D ground truth as it is intrinsically not available in 2D MR images.

This work advances from heuristic methods and systematically investigates the impact of five different 2D cine MR imaging parameters on the ability of template-matching-driven autocontouring to localize anatomical structures in three dimensions. This is facilitated using artificial 2D cine MR images, generated from 4D MR scans. This novel approach allows us to create 2D images at many different imaging settings and provides us with a matched 3D ground truth contour to benchmark all investigated settings. Furthermore, by using specifically acquired repeat 4D MR scans, we were able to not only explore the effect of periodic respiratory motion, but also of slow baseline drifts or deformations of the tumor over the course of several minutes.

## 2. Materials and methods

In this study, we used 4D MR images (defined as multiple 3D MR images at ten retrospectively determined breathing phases) of 14 different subjects, six healthy volunteers and eight lung cancer patients (see section 2.1). These 4D MR images were used to generate artificial 2D cine MR images, while simultaneously serving as ground truth (see sections 2.2 and 2.3). We created training or test images to systematically explore the impact of five different imaging parameters, which are listed in table 1. Three parameters, ‘pixel size’, ‘slice thickness’ and ‘imaging plane orientation’, describe the 2D cine MR images themselves. Another parameter, ‘acquisition strategy’, governs the number and relative alignment of the 2D cine MR images. The final parameter, ‘template generation strategy’, describes the training images used to train a template-matching algorithm. The algorithm was used to automatically localize either the left kidney and gallbladder or the lung tumor (see section 2.4). By comparing the automatically determined contours to the ground truth contours via centroid distance and Dice similarity coefficient, we were able to assess the impact of the different imaging parameters on the localization accuracy (see section 2.5).

**Table 1.** List of imaging parameters investigated in this study. The centroid position of the volume of interest was defined in the peak-exhale phase of the first scan and determined how all generated 2D images were positioned.

Imaging parameter	Investigated settings	Description
Pixel size	2.0 mm–5.0 mm in 1.0 mm steps	Square pixels

Slice thickness	5.0 mm–10.0 mm in 2.5 mm steps	

Imaging plane orientation	−80° to 90° in 10° steps	Rotation around the superior–inferior axis, rotation angle relative to the coronal plane

Acquisition strategy	*Slice*	A single plane intersecting with the centroid of the volume of interest
	*Stack*	Three parallel, adjacent imaging planes with the central one passing through the tumor or organ centroid
	*Cross*	Two orthogonal planes, intersecting at the tumor or organ centroid

Template generation strategy	*2Dsingle*	A single template per imaging plane is extracted from the peak-exhale phase of the first scan
	*2Dmulti*	10 templates are created from all respiratory phases of the first scan
	*3Dsingle*	2 additional templates (3 in total) are generated from adjacent imaging planes from the peak-exhale phase, potentially increasing robustness against through-plane motion
	*3Dmulti*	Similar to *3Dsingle*, 20 additional templates (30 in total) are extracted from the adjacent slices at all 10 respiratory phases

### 2.1. Acquisition and reconstruction of 4D MR images

For this study, we acquired repeat 4D MR scans of the upper abdomen of six healthy volunteers. Additionally, we used thoracic 4D MR scans of eight patients undergoing treatment for non-small cell lung cancer (NSCLC) at our institution. Scanning was approved by our institutional review board and all volunteers and patients consented to the use of their images for research purposes. All scans were acquired with a 1.5 T MAGNETOM Aera scanner (Siemens Healthcare, Erlangen, Germany) using a T1-weighted, stack-of-stars, golden-angle, radial spoiled gradient-echo sequence (Block *et al* 2014). The 4D MR scans were reconstructed at ten respiratory phases each using the joint MoCo-HDTV algorithm (Rank *et al* 2017). Details regarding the sequence and reconstruction parameters are provided in table 2.

**Table 2.** Settings of the T1-weighted, stack-of-stars, golden-angle, radial spoiled gradient-echo sequence used in this study to obtain abdominal 4D MR scans of six healthy volunteers and thoracic 4D MR scans of eight lung cancer patients. The scans were reconstructed using the joint MoCo-HDTV algorithm (Rank *et al* 2017).

	Abdominal scans—all volunteers	Thoracic scans—patients 1–3	Thoracic scans—patients 4–5	Thoracic scans—patients 6–8
Repetition time (ms)	3.99	2.50	3.18	3.18
Echo time (ms)	2.39	1.25	1.57	1.57
Flip angle (°)	12	12	8	8
Slice thickness (mm)	3.0	3.5	3.3	3.0
Pixel bandwidth (Hz)	630	1085	630	630
Acquisition time (min)	(3×) 5:08	5:45	5:45	5:45
Reconstructed voxel size (mm^3^)	$1.25\times1.25\times1.5$	$1.5\times1.5\times1.5$	$1.25\times1.25\times1.75$	$1.25\times1.25\times1.65$
Additional comments	Out-of-phase contrast	Fat suppression	Fat suppression	Fat suppression

**Figure 1.**
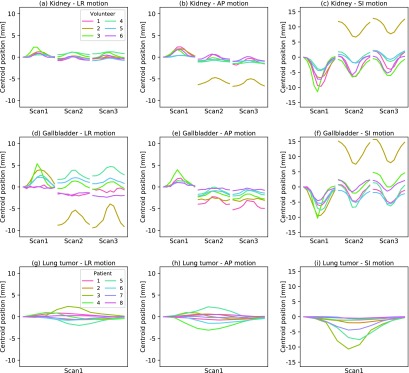
Observed centroid motion of the (a)–(f) left kidney and gallbladder in the repeat 4D MR scans of the six healthy volunteers and (g)–(i) lung tumor in the single 4D MR scan of the eight patients used in this study. Shown is the shift of the centroid at each of the ten breathing phases per scan measured in left–right (LR), anterior–posterior (AP) and superior–inferior (SI) direction, respectively, relative to the peak-exhale phase of the first scan.

Each healthy volunteer was scanned three times with 5–10 min breaks in between scans during which the volunteers remained on the scanner couch. While the volunteers were asked to remain still with their arms down for the entire imaging session, no breathing instructions were given or special fixation was used. The acquired imaging dataset is able to resolve both periodic respiratory-induced motion as well as baseline drifts due to changes in respiratory pattern and gastrointestinal movement (see figures 1(a)–(f)). In five out of six healthy volunteers, the measured motion of the kidney and gallbladder was similar to that reported for cancer patients (Shirato *et al* 2004, Sonier *et al* 2016). In volunteer 2, the kidney and gallbladder both shifted by 15 mm in between the first and second repeat MR scan. The subject may have altered its position and breathing after the first MR scan, although we did not observe large movement of the bony anatomy. It is palpable that the use of radiotherapy fixation equipment would have mitigated this very large shift.

We also used the 4D MR images of eight NSCLC patients with a localized primary tumor (primary tumor stage  ⩽  T2 according to the American Joint Committee on Cancer’s guidelines on lung cancer staging, 7th edition). The scans were acquired in radiotherapy treatment position, but without any specific breathing instructions being provided. While the single 4D MR scans of the lung cancer patients cannot inform us about potential baseline drifts, the images contained lung tumors influenced by periodic respiratory motion (see figures 1(g)–(i)). The observed lung tumor motion magnitude was comparable to previously reported values (Shirato *et al* 2004, Knybel *et al* 2016). Additional details regarding the scanned volunteers (organ size) and patients (tumor staging, position and size) can be found in the supplemental material (see appendix A, tables A1 and A2).

### 2.2. Contouring and generation of 3D ground truth

For the healthy volunteer 4D MR scans, we used the left kidney and gallbladder as volumes of interest throughout the study. We chose these two anatomical structures as they were recognizable in the T1-weighted MR images and exhibited movement patterns influenced by both periodic breathing motion and baseline drifts. The organs were delineated in the peak-exhale image of the first scan by a single observer. Then, ADMIRE, research version 1.14.1.1 (Elekta AB, Stockholm, Sweden) was used to deformably register this image to the other 29 images of the same volunteer and propagate the contours. The two-step deformable image registration first uses a block-matching registration driven by a mutual information similarity metric. In the second step, the derived deformable vector field is further optimized using a free-form deformable registration driven by a combination of mutual information and the sum-of-squared-differences. For the patient 4D MR scans, the lung tumor was contoured in the peak-exhale image and the contours were propagated to the remaining nine images. All contours were visually verified before proceeding. In the following, these images and contours served as 3D ground truth throughout the study and were used to generate artificial 2D cine MR images.

### 2.3. Generation of artificial 2D cine MR images

Artificial 2D cine MR images were created using MATLAB, version R2017a (MathWorks, Natick, MA, USA). Initially, the 3D contours were transformed into a binary 3D contourmask using MATLAB’s poly2mask function. Next, the 2D images and contourmasks at any designated image resolution, position and orientation were populated using tri-linear interpolation of the 3D images and contourmasks. Supersampling was used to avoid aliasing artifacts.

Due to the substantially longer imaging time and deployed reconstruction method of the 4D MR scans, the artificial 2D cine MR images featured considerably less image noise than expected in real 2D cine MR images. Noise in MR images is primarily caused by resistance of the MR scanner’s receiving coil system as well as dielectric and inductive losses in the subject (Gudbjartsson and Patz 1995). It can be characterized by a Rician probability density function:

1 \begin{align*} \newcommand{\e}{{\rm e}} \displaystyle \label{eq:rician} P(x) = \frac{x}{\sigma^2} \; {\rm exp}\Bigg(\frac{-x^2-a^2}{2\sigma^2}\Bigg) \; I_0\Bigg(\frac{xa}{\sigma^2}\Bigg), \nonumber \end{align*}

with the scale of the noise *σ*, the true image intensity before addition of the noise *a* and *I*_0_(*x*) denoting the modified Bessel function of the first kind. Ultimately, the image’s signal-to-noise-ratio SNR depends on the acquisition matrix’s field of view in frequency-encoding direction $FOV_x$ and phase-encoding direction $FOV_y$, the matrix’s size in frequency-encoding direction *N*_*x*_ and phase-encoding direction *N*_*y*_, as well as the imaging slice thickness *z* and pixel bandwidth *BW* (McRobbie *et al* 2017):

2 \begin{align*} \newcommand{\e}{{\rm e}} \displaystyle \label{eq:noise_scaling} {\rm SNR} \propto \frac{FOV_x}{N_x}\frac{FOV_y}{N_y}{z} \; \sqrt{\frac{N_x N_y}{BW N_x}}. \nonumber \end{align*}

The noise scale *σ* of equation (1) was estimated in experimentally acquired 2D cine MR images using two strategies. Both strategies used images of a healthy volunteer that were obtained using a T1-weighted spoiled gradient-echo sequence (repetition time: 3.99 ms, echo time: 2.39 ms, flip angle: 12°, field-of-view: $320\times320$ mm^2^, acquisition matrix size: $160\times160$, slice thickness: 5 mm, pixel bandwidth: 1080 Hz). The first approach is based on the Rician distribution simplifying to a Rayleigh distribution:

3 \begin{align*} \newcommand{\e}{{\rm e}} \displaystyle \label{eq:rayleigh} P(x) = \frac{x}{\sigma^2} \; {\rm exp}\Bigg(\frac{-x^2}{2\sigma^2}\Bigg) \nonumber \end{align*}

in areas without signal (*a*  =  0). The variance $v$ of equation (3) is given by:

4 \begin{align*} \newcommand{\e}{{\rm e}} \displaystyle \label{eq:rayleigh_variance} v = \frac{4 - \pi}{2}\sigma^2. \nonumber \end{align*}

By measuring the variance of the image signal in air surrounding the subject, we can calculate *σ*. However, the combined image from multiple receiving coils may have suppressed image noise in air (Roemer *et al* 1990). The second approach exploits the fact that for large pixel intensities, a Gaussian distribution can be used to approximate the Rician distribution (Gudbjartsson and Patz 1995). We determined the temporal variance of single voxels in the volunteer’s liver over several subsequently acquired 2D cine MR images and used its square root to calculate *σ*.

**Figure 2.**
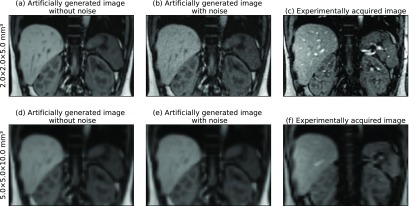
Side-by-side comparison of artificially created images (with and without added Rician noise) and experimentally acquired 2D cine MR images at (a)–(c) high and (d)–(f) low image resolution. Despite some differences in contrast, especially in blood or bony anatomy, the soft-tissue contrast and signal-to-noise ratio is comparable in artificial and real images.

Ultimately, we used the average *σ* value from both approaches to add Rician noise to the artificial images (see figure 2). Thereby, the noise level determined at a resolution of $2\times2\times5$ mm^3^ was scaled according to equation (2) for images created at lower resolutions.

### 2.4. Automated organ and tumor localization based on template matching

**Figure 3.**
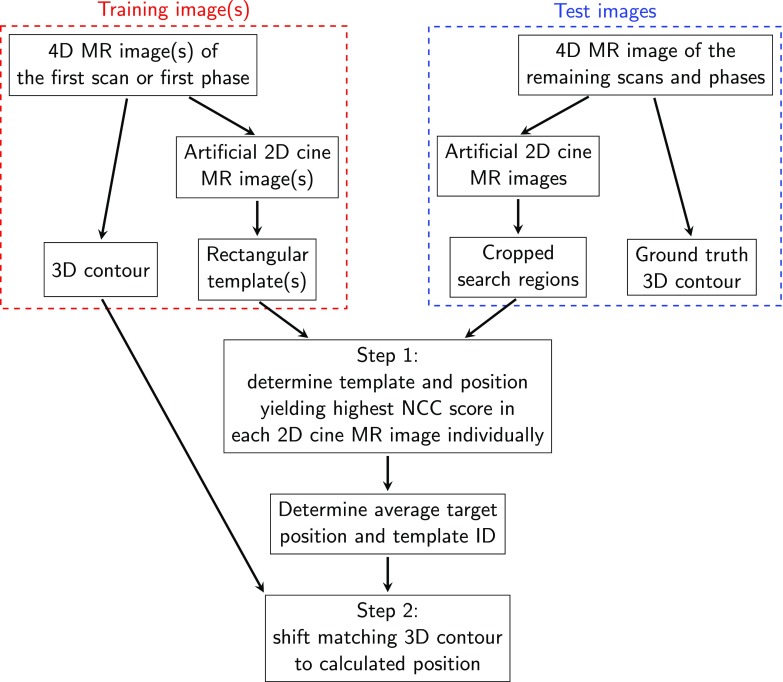
Schematic presenting the workflow to automatically determine the 3D contour. The number of used 2D cine MR images and templates is governed by the acquisition strategy and template generation strategy, respectively.

Automated localization of the volumes of interest was achieved in two steps (see figure 3). First, the tumor or organ position was identified in each 2D imaging plane independently (one plane for *slice* acquisition strategy, three for *stack* strategy, two for *cross* strategy). This was achieved using a previously developed template matching algorithm (Fast *et al* 2017). Our version is implemented in C++ and able to determine the target position in less than 1 ms on a standard desktop computer (Intel Core i7-6720 CPU at 2.7 GHz (Intel Corporation, Santa Clara, CA, USA)). It calculates the normalized cross correlation score of one or multiple rectangular templates at each possible position in a search region, which spans the maximum extent of the volume of interest in the peak-exhale phase of the first scan plus a 2.5 cm margin in each direction. The template and position yielding the highest normalized cross correlation score are used in the following.

In the second step, the information obtained via template matching is used to reposition a 3D contour. For kidney and gallbladder autocontouring, this 3D contour is extracted from the first repeat 4D MR scan, while the peak-exhale contour of the single 4D MR scan is used when locating the lung tumors. For the *slice* acquisition strategy, the 3D contour belonging to the best matching template is moved according to the detected target shift. For the *stack* and *cross* strategy, the positions determined in each imaging plane are averaged and the 3D contour of the template used by the primary imaging plane (central plane for *stack* strategy, first plane for *cross* strategy) is shifted to this position.

### 2.5. Evaluation of 3D localization accuracy

For each possible combination of imaging parameters, we automatically contoured the volumes of interest in all test images. The adapted 3D contours were compared to the ground truth via the centroid distance (CD), defined as the euclidean distance between the centroid of both contours in three dimensions, and Dice similarity coefficient (DSC), defined as two times the union of the volumes divided by the sum of both volumes. Similarly, CD and DSC were calculated for the static peak-exhale and mid-ventilation contour extracted from the first scan, emulating a non-adaptive treatment strategy.

For the left kidney, 311 040 data points (2592 combinations of imaging parameters  ×  6 volunteers  ×  2 scans  ×  10 breathing phases) were included in the analysis. For the gallbladder, the number of data points was reduced to 156 520 (1296 combinations of imaging parameters  ×  6 volunteers  ×  2 scans  ×  10 breathing phases) as we had to exclude the *3Dsingle* and *3Dmulti* template generation strategies because the adjacent template planes commonly extended beyond the small gallbladder. For the same reason, we had to exclude the *3Dsingle* strategy when locating lung tumors. Template generation strategies using all breathing phases (*2Dmulti* and *3Dmulti*) were intrinsically not testable with only a single 4D MR scan available per lung cancer patient. Overall, we acquired 46 656 data points (648 combinations of imaging parameters  ×  8 patients  ×  1 scan  ×  9 breathing phases) while automatically delineating lung tumors.

We compared the observed distribution of the CD and DSC data points dependent on the five different imaging parameters. This allowed us to evaluate their impact on the localization accuracy. Additionally, we investigated the dependency of the distribution on the imaging plane orientation relative to the main motion direction in the axial plane (as all images were orientated along the superior–inferior axis). The main motion direction was determined via principal component analysis of the 3D centroid motion of the volume of interest in the first 4D MR scan. In order to assess the dependency of the localization accuracy on varying imaging contrast and motion magnitude, we also evaluated inter-subject variations in the CD and DSC distributions. Additionally, we compared the CD and DSC of the static peak-exhale or mid-ventilation contours to the metrics for the adapted contours.

## 3. Results

**Figure B1.**
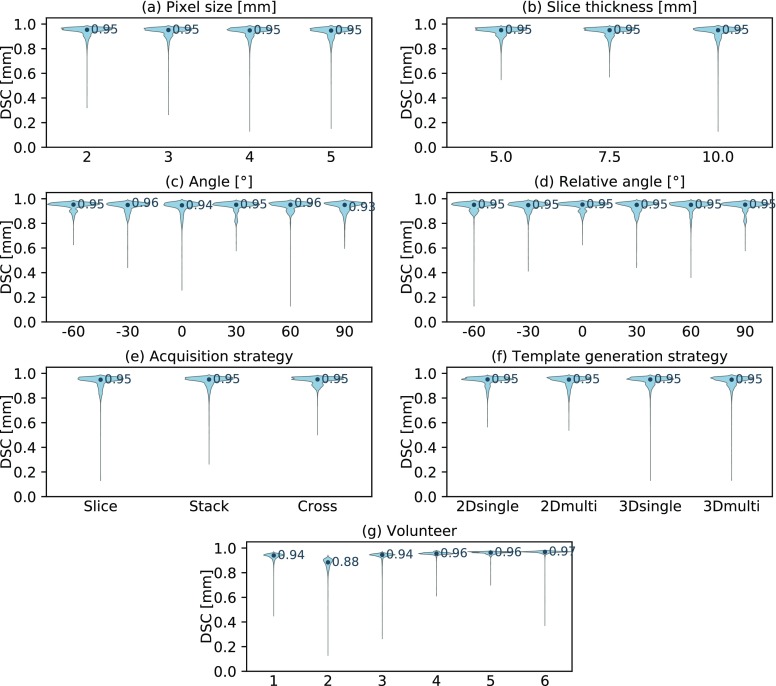
Violin plots presenting the distribution of Dice similarity coefficient (DSC) between the adapted and ground truth kidney contour. (a)–(g) Each plot showcases the dependency of the distribution on a specific imaging parameter. Note that some parameter settings are omitted for the ‘angle’ and ‘relative angle’ plots in order to increase their readability.

**Figure B2.**
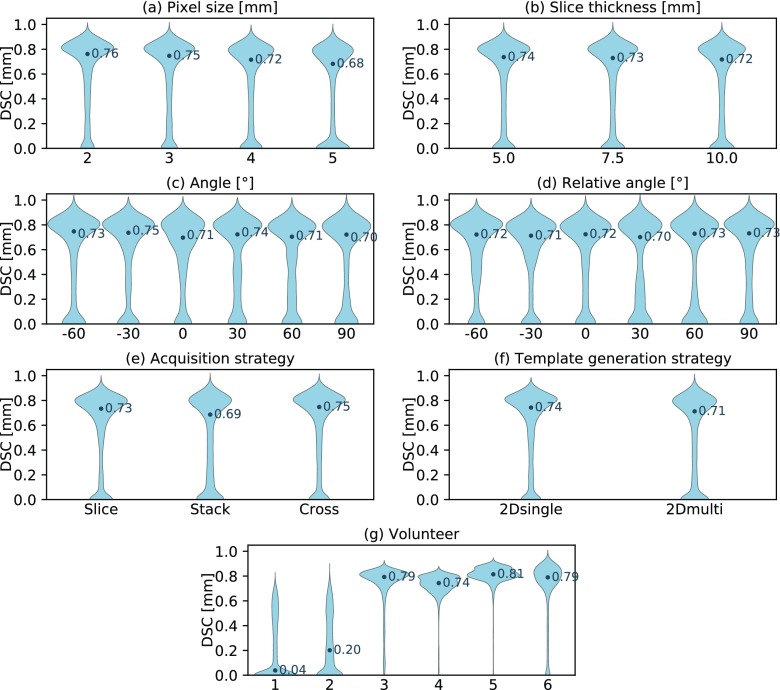
Violin plots presenting the distribution of Dice similarity coefficient (DSC) between the adapted and ground truth gallbladder contour. (a)–(g) Each plot showcases the dependency of the distribution on a specific imaging parameter. Note that some parameter settings are omitted for the ‘angle’ and ‘relative angle’ plots in order to increase their readability.

**Figure B3.**
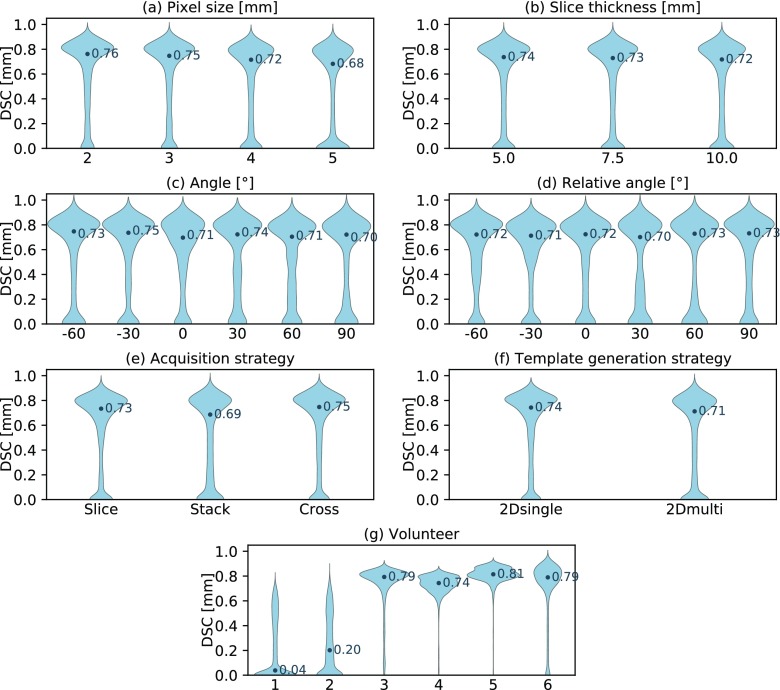
Violin plots presenting the distribution of Dice similarity coefficient (DSC) between the adapted and ground truth lung tumor contour. (a)–(g) Each plot showcases the dependency of the distribution on a specific imaging parameter. Note that some parameter settings are omitted for the ‘angle’ and ‘relative angle’ plots in order to increase their readability.

For all three investigated volumes of interest, the automatically determined contours were more accurate than either the peak-exhale or mid-ventilation contours (see table 3). The following sections present the dependency of the localization accuracy on the different imaging parameters for the left kidney (section 3.1), gallbladder (section 3.2) and lung tumor (section 3.3). In addition to images and contours from representative example cases, violin plots are used to illustrate the results. Each ‘violin’ displays the distribution of the data points measured for a specific imaging parameter. Violin width is governed by the probability density of the data points. By displaying multiple violins with a varied imaging parameter next to each other, it is possible to assess that parameter’s impact. Additionally, we denote the median of each distribution with a labeled dot. As the CD and DSC were found to be highly correlated (Pearson coefficient:  −0.91 for kidney, −0.84 for gallbladder, −0.94 for lung tumor), we only show and discuss results using CD as accuracy metric. The DSC-based violin plots are included in the supplemental material (see appendix B, figures B1, B2 and B3).

**Table 3.** Comparison of the accuracy of the adapted contours with the static peak-exhale (PE) and mid-ventilation contours (MV) via centroid distance (CD) and Dice similarity coefficient (DSC). 5th and 95th denote the 5th and 95th percentile, respectively. All values are averaged over all investigated combinations of imaging parameters. Kidney and gallbladder contours were compared to the ground truth contours for all phases of the second and third scan, while the lung tumor contours were compared to the contours of the eight breathing phases of the single scan other than the peak-exhale and mid-ventilation phase.

		CD (mm)			DSC		
		5th	Median	95th	5th	Median	95th
Kidney	Adapted	0.70	1.56	6.30	0.85	0.95	0.97
	PE	0.88	2.13	12.99	0.76	0.94	0.97
	MV	1.15	4.94	18.05	0.68	0.91	0.96

Gallbladder	Adapted	1.21	3.81	20.77	0.00	0.72	0.87
	PE	1.97	3.94	17.00	0.00	0.70	0.83
	MV	2.09	5.63	24.24	0.00	0.52	0.81

Lung tumor	Adapted	0.22	1.03	4.72	0.67	0.93	0.98
	PE	0.19	1.13	7.69	0.55	0.93	0.98
	MV	0.19	1.22	6.36	0.65	0.92	0.98

### 3.1. Kidney localization accuracy dependent on imaging parameters

**Figure 4.**
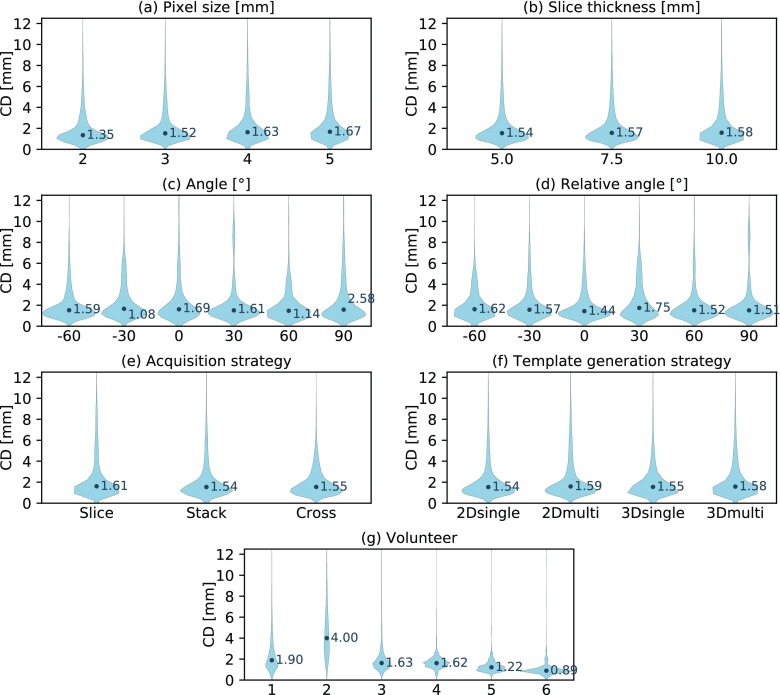
Violin plots presenting the distribution of centroid distances (CD) between the adapted and ground truth kidney contour. (a)–(g) Each plot showcases the dependency of the distribution on a specific imaging parameter. Note that some parameter settings are omitted for the ‘angle’ and ‘relative angle’ plots in order to increase their readability.

**Figure 5.**
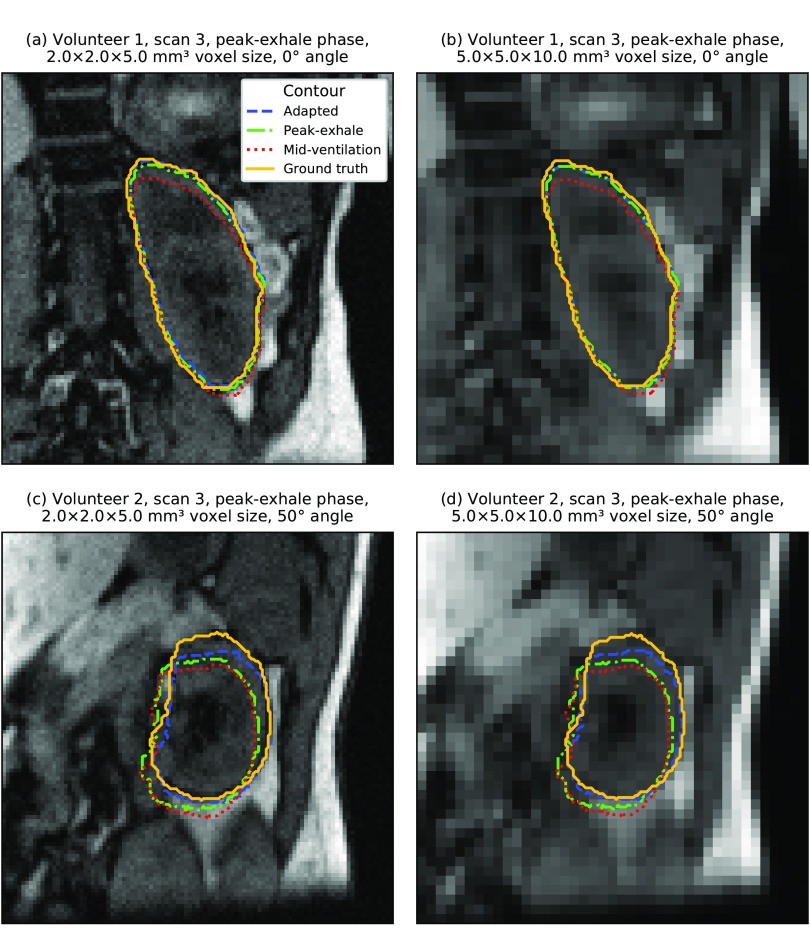
(a)–(d) Artificially generated 2D cine MR images. Overlaid are the ground truth and adapted kidney contours as well as the static peak-exhale and mid-ventilation contours from the first scan. In all examples, the *slice* acquisition strategy and *2Dsingle* template generation strategy were used. These examples illustrate the limited impact of image resolution on localization accuracy for the kidney.

Most noteworthy, the imaged volunteer had a substantially larger impact on localization accuracy than any of the deployed imaging parameters (see figure 4(g)). Of the geometric imaging parameters, only the pixel size had a modest impact on localization accuracy (see figure 4(a)). Still, in many cases comparable localization errors were scored despite using images of much lower resolution (see figure 5). In several volunteers we observed favorable imaging plane orientations. However, averaged over all volunteers, we were not able to explain these findings by means of the absolute plane angle (see figure 4(c)) or angle relative to the main motion direction (see figure 4(d)).

### 3.2. Gallbladder localization accuracy dependent on imaging parameters

**Figure 6.**
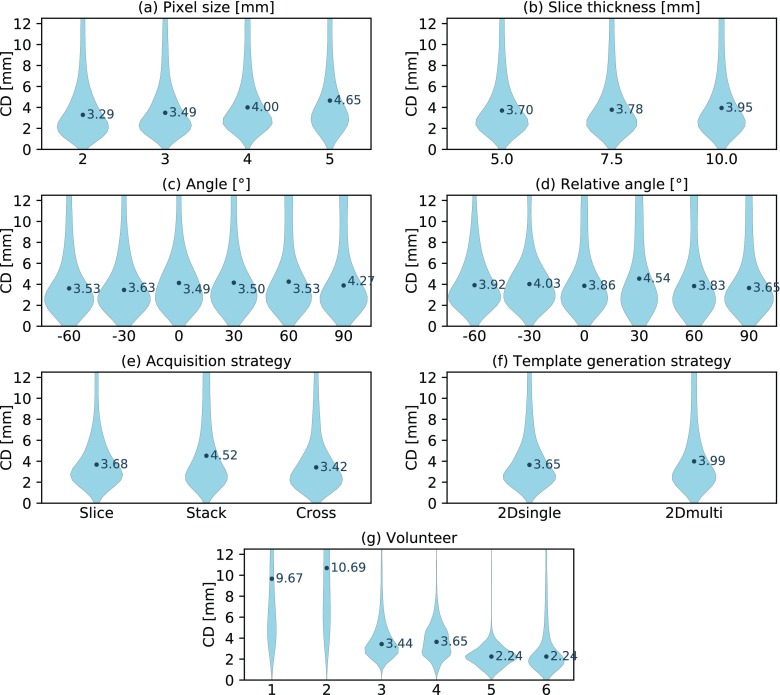
Violin plots presenting the distribution of centroid distances (CD) between the adapted and ground truth gallbladder contour. (a)–(g) Each plot showcases the dependency of the distribution on a specific imaging parameter. Note that some parameter settings are omitted for the ‘angle’ and ‘relative angle’ plots in order to increase their readability.

**Figure 7.**
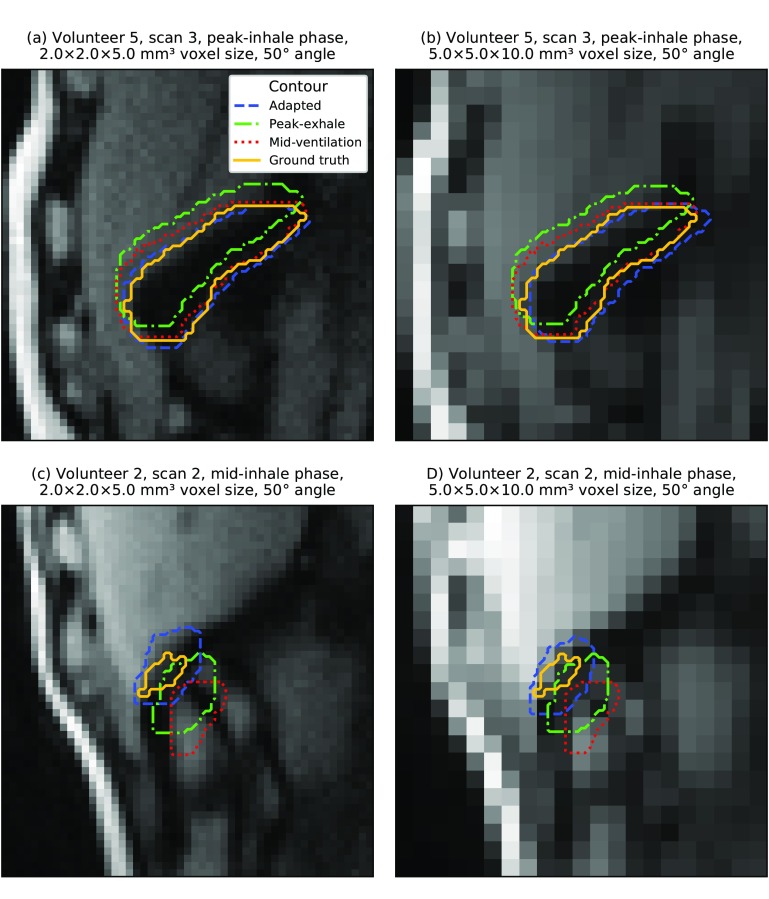
(a)–(d) Artificially generated 2D cine MR images. Overlaid are the ground truth and adapted gallbladder contours as well as the static peak-exhale and mid-ventilation contours from the first scan. In all examples, the *slice* acquisition strategy and *2Dsingle* template generation strategy were used. Localization accuracy for the gallbladder depended more on the gallbladder size and image contrast than on image resolution.

The localization accuracy for the gallbladder was lower than for the kidney, although the adapted contours were still more accurate than the static ones (see table 3). The localization error increased for larger pixel sizes (see figure 6(a)), larger slice thicknesses (see figure 6(b)) and dependent on the acquisition strategy (see figure 6(e)). It appears that the small size of the gallbladder is challenging for the *stack* imaging strategy at large slice thicknesses. In these cases, the outer imaging slices commonly only capture a small cross section of the gallbladder causing a worse template matching performance. Again, inter-subject variations had the largest impact on localization accuracy with an even more pronounced effect for the gallbladder than for the kidney (see figure 6(g)). Volunteer 1 and 2 both featured a very small gallbladder during imaging and their images exhibited a poor contrast, leading to a substantially worse localization accuracy compared to the other four subjects (see figure 7).

### 3.3. Lung tumor localization accuracy dependent on imaging parameters

**Figure 8.**
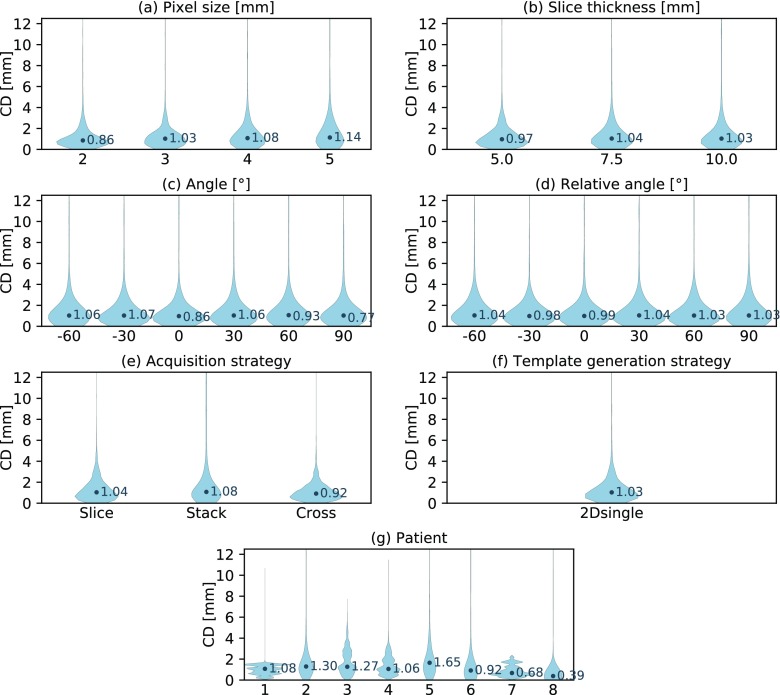
Violin plots presenting the distribution of centroid distances (CD) between the adapted and ground truth lung tumor contour. (a)–(j) Each plot showcases the dependency of the distribution on a specific imaging parameter. Note that some parameter settings are omitted for the ‘angle’ and ‘relative angle’ plots in order to increase their readability.

**Figure 9.**
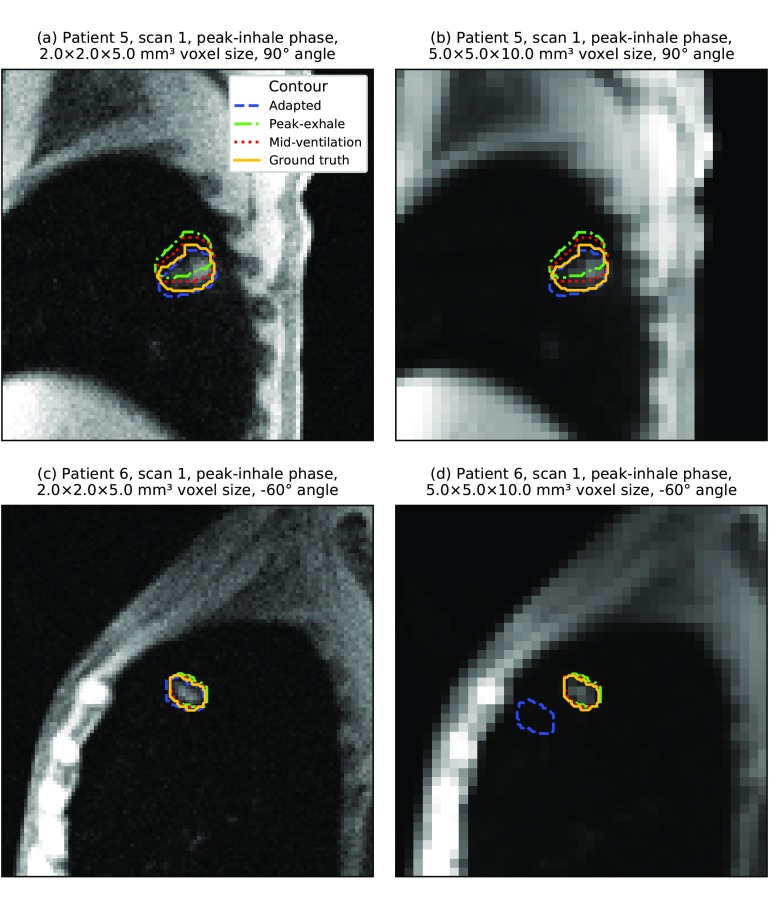
(a)–(d) Artificially generated 2D cine MR images. Overlaid are the ground truth and adapted lung tumor contours as well as the static peak-exhale and mid-ventilation contours from the first scan. In all examples, the *slice* acquisition strategy and *2Dsingle* template generation strategy were used. For very small tumors, localization sometimes failed using low-resolution images due to the tumor-to-background-contrast.

The template matching algorithm was able to delineate lung tumors with the highest accuracy of all three volumes of interest (see table 3). However, it has to be noted that on average the lung tumors also exhibited the smallest motion magnitude and baseline drifts were intrinsically not included in the dataset. The lack of observed motion in the axial plane also reduced the informative value of the slice thickness and image orientation analysis. Out of all geometric imaging parameters, changes in pixel size affected localization accuracy the most (see figure 8(a)). When localizing very small tumors in low-resolution images, we sometimes observed the template matching algorithm failing to find the correct position (see figure 9). However, this occurred less frequently than for the gallbladder. Once again, inter-subject variations had the largest impact (see figure 8(g)).

## 4. Discussion

The most prominent finding of this study is the robustness of template-matching-based tumor and organ localization against varying geometric imaging parameters. Even the most pronounced trend, a two-and-a-half-fold increase in pixel size, only had a limited effect compared to the inter-subject variations. Similarly, robustness to different imaging parameters of their—admittedly more complex—particle-filter-driven autocontouring algorithm was reported by Bourque *et al*, when testing their algorithm on 18 2D cine MR imaging sets acquired at varying imaging settings (Bourque *et al* 2018).

We did not observe an increase in accuracy when providing the template matching algorithm with training images from several respiratory phases (*2Dmulti* versus *2Dsingle* and *3Dmulti* versus *3Dsingle* template generation strategy). However, in the eight imaged volunteers, the volumes of interest did not substantially deform or rotate with periodic breathing. For strongly deforming targets, such as cardiac structures or some tumors, using multiple templates may reduce contouring errors in the context of template matching algorithms (Ipsen *et al* 2016).

Including the templates from adjacent imaging planes (*3Dsingle* versus *2Dsingle* and *3Dmulti* versus *2Dmulti* template generation strategy) also did not improve localization accuracy. It should be noted that in this study all imaging planes were orientated in superior–inferior direction, in which the largest motion is expected. A study by Brix *et al* reported that using multiple 2D templates generated from a previously acquired 3D MR scan allowed accurate estimation of through-plane motion of capillary phantom and liver structures (Brix *et al* 2014). It seems that anatomical changes in between the first scan, used to create templates, and the subsequent scans, used to generate test images, neutralize any expected benefit of using multiple templates.

The high inter-subject variation of the localization accuracy suggests that other, non-geometric imaging parameters govern it. We suspect that both the magnitude of the encountered motion as well as the observed contrast play an important role. Indeed, volunteer 2, for whom the worst localization results were obtained, featured relatively poor image contrast and the largest observed organ motion. As all volunteer images in this study were acquired at an out-of-phase fat-water contrast, the fraction of visceral fat had a large influence on organ visibility. Additionally, contrast of the monitored structure and its surrounding depended on the size of the volume of interest as well as proximity and relative location of other adjacent organs. These factors can also explain the observed occurrences of subject-specific plane orientations that yielded better results.

In the context of real-time adaptive radiotherapy, these results suggest that only moderate accuracy gains are to be expected from increasing imaging resolution or number of imaging planes. Relinquishing either might facilitate an increased imaging rate. Template matching algorithms, for example, could benefit from this by using a smaller, dynamically updated search region. In addition to fine-tuning automated contouring algorithms, radiotherapy research should focus on optimizing imaging contrast. Both tasks may be specific to imaged anatomical site or even patient.

This study did not compare different MR sequences for real-time adaptive radiotherapy. All results are based on T1-weighted spoiled gradient-echo 4D MR acquistions to provide us with high-quality, artifact-free base images. As shown in section 2.3, the simulated 2D cine MR images featured a realistic contrast and signal-to-noise-ratio, similar to what is achievable with a 2D spoiled gradient-echo acquisition. Still, some differences in the presence of blood or bones remain. Additionally, several physical aspects of MR imaging, such as non-linearities of the *B*_0_ and gradient magnetic field, the finite duration of the slice excitation pulse, the dependency of the image noise on receiving coil setup and cancelling out of the imaging signal in regions of slice overlap are not considered during image generation.

Another shortcoming of this study is the deployed methodology to derive the training and ground truth contours. We manually outlined the respective volumes of interest on a single 4D MR image phase and used deformable image registration to propagate the contours to all other respiratory phases and repeat scans. Even though visual inspection found the contours to be sufficiently accurate, residual errors due to inter-observer variability and deformable image registration algorithm may remain (Kaus *et al* 2007, Louie *et al* 2010). However, we do not think that these errors meaningfully impact the comparison of the different imaging parameters, the main goal of this study. Alternatively, a digital phantom could be used to generate medical images with the ground truth tumor and organ volumes intrinsically available (Segars *et al* 2010). Additionally, this approach allows simulation of a large number of different anatomies and anatomical changes, although digital phantoms are still limited with regard to anatomical detail and realism of the simulated imaging process (Lowther *et al* 2018).

Ultimately, we believe that the ability to create 2D cine MR images of 14 human subjects at freely selectable geometric imaging parameters outweighs these shortcomings. To our knowledge, this is the first study looking into automated tumor organ localization with such a large imaging parameter space and reporting localization accuracy results based on a matched 3D ground truth. Additionally, the repeat 4D MR scans of the healthy volunteers allowed us to resolve baseline variations in patient anatomy, which remains an underappreciated problem in radiotherapy (Menten *et al* 2016, Dhont *et al* 2017).

## 5. Conclusions

This study has found that automated tumor and organ localization using 2D cine MR imaging and template-matching-based autocontouring is robust against varying geometric imaging parameters. Future work and optimization efforts of MR imaging for real-time adaptive radiotherapy is needed to characterize the influence of sequence- and anatomical-site-specific imaging contrast.

## Appendix A. Additional details of imaged volunteers and patients

**Table A1.** Kidney and gallbladder size of the healthy volunteers imaged for this study. The organ size was determined in the peak-exhale phase of the first 4D MR scan.

Volunteer	Kidney size (cm^3^)	Gallbladder size (cm^3^)
1	156.4	1.9
2	155.6	2.0
3	149.6	6.4
4	173.0	12.2
5	162.5	10.9
6	146.2	4.3

**Table A2.** Characteristics of the lung cancer patients used in this study. Staging according to American Joint Committee on Cancer’s guidelines on lung cancer staging, 7th edition. LUL, LLL, RUL, RML and RLL denote left upper lobe, left lower lobe, right upper lobe, right middle lobe and right lower lobe, respectively. Tumor size is based on the peak-exhale contour.

Patient	TMN stage	Tumor location	Tumor size (cm^3^)
1	T2N0M0	LUL	10.6
2	T2N2M0	RML	6.6
3	T2N1M0	LLL	25.6
4	T1N0M0	LUL	8.1
5	T2N2M0	RLL	5.1
6	T1N0M0	RUL	3.7
7	T2N0M1	LLL	44.9
8	T1N0M0	LUL	2.1
